# Impact of Pesticide Use on Gut Microbiota and Health: A Systematic Review of Findings in Both Humans and Animal Models

**DOI:** 10.3390/jox16020041

**Published:** 2026-02-25

**Authors:** Iria Osa-Subtil, Teolincacihuatl Romero-Rosales, María José Dios-Duarte

**Affiliations:** 1 Department of Medicine, Faculty of Medicine, Health and Sports, Universidad Europea de Madrid, 28670 Villaviciosa de Odón, Spain; iria.delaosa@universidadeuropea.es; 2 Psychosocial Factors and Social Intervention (Research Group), Complutense University of Madrid, 28223 Madrid, Spain; 3 Faculty of Agricultural and Environmental Sciences, Autonomous University of Guerrero, Iguala de la Independencia 40000, Mexico; 18029@uagro.mx; 4 Nursing Department, Faculty of Nursing, University of Valladolid, 47005 Valladolid, Spain

**Keywords:** pesticide exposure, human health, animal health, gut microbiota, dysbiosis, diseases

## Abstract

**Background/objective:** The widespread use of pesticides in modern agriculture has raised increasing concern about their potential adverse effects on human health. Exposure to these compounds has been linked to multiple negative health outcomes. This systematic review aims to evaluate and synthesise the available scientific evidence on the effects of pesticide exposure on human health during agricultural production—with particular emphasis on alterations in gut microbiota and intestinal membrane permeability—by integrating results from experimental and observational studies conducted on animals and humans. **Methods:** This systematic review was conducted in accordance with PRISMA guidelines. A systematic literature search was carried out using the main databases Medline/PubMed, Embase and Web of Science, introducing the search algorithm “pesticides” AND “gut microbiota”, from which a total of seven systematic reviews that met our inclusion criteria were found and subsequently analysed. The quality assessment was based on the principles of evidence-based medicine. This systematic review was registered in the OSF. **Results:** The findings indicate that prenatal exposure to pesticides is linked to adverse outcomes in foetal development. Additionally, pesticide exposure affects metabolic, immune, and nervous system function due to alterations in gut microbiota composition and membrane permeability. Evidence from animal model studies complements human data by providing insight into the underlying biological mechanisms, such as oxidative stress, liver dysfunction, alterations in hormonal signalling and activation of the inflammatory response. **Conclusions:** Public health strategies should prioritise reducing pesticide exposure, strengthening environmental protection and supporting further research on gut microbiota modulation and intestinal membrane permeability. Such measures may contribute to the prevention and mitigation of pesticide-related health disorders. **Limitations:** Human data are insufficient to establish clear causal relationships. Moreover, substantial variability among pesticide types and the difficulty of distinguishing the effects of complex mixtures from those of individual compounds complicate interpretation of the findings.

## 1. Introduction

Pesticides are natural or synthetic chemical substances used to eradicate pests and insects, and are essential for improving agricultural productivity [1,2]. They increase crop yields and protect plants from disease and damage. Pesticides are divided into several different categories such as herbicides, insecticides, fungicides, molluscicides, ovicides, acaricides, rodenticides and nematicides, with fungicides, insecticides and herbicides being the most widely used [1,2,3,4]. The extensive use of pesticides represents one of the primary sources of toxic compound exposure in the general population, posing a significant environmental toxicology problem [2,3,5]. These chemical agents are designed to eliminate organisms that are considered harmful [5]. However, they can interfere with multiple biological processes in humans, particularly if exposure is chronic or involves combined exposure to multiple products [6,7,8]. Numerous studies have demonstrated that pesticide toxicity occurs via mechanisms such as oxidative stress, altered cholinergic neurotransmission, endocrine disruption, and genotoxic damage [9,10,11,12]. The consequences of pesticide exposure are wide-ranging. They can include acute effects such as respiratory or neuromuscular toxicity, as well as chronic conditions associated with neurodegenerative pathologies and reproductive disorders [2,5]. Studies have also documented the cumulative toxic effects of pesticides on human health and non-target organisms, as well as their capacity for bioaccumulation, which is associated with endocrine disruption and the development of chronic diseases in exposed populations [2,13].

Advances in molecular biology and microbiology have highlighted the critical roles of intestinal permeability and the gut microbiota in systemic health and disease development [14,15]. The gut microbiota plays a fundamental role in maintaining homeostasis and human health by performing protective, structural and metabolic functions essential for physiological balance, including the production of bioactive metabolites, immune regulation and maintenance of epithelial barrier integrity [16,17]. The intestinal mucosal layer acts as the body’s first line of defence by regulating mucus secretion and bacterial degradation, thereby limiting exposure to antigens and pro-inflammatory molecules [18]. In addition, specific bacterial communities reinforce the tight junctions between epithelial cells, preventing macromolecules and endotoxins from entering into the bloodstream [19,20]. The gut microbiota principally contributes to modulating the immune system, regulating the gut–brain axis, synthesising vitamins and bioactive metabolites, protecting the intestinal barrier, and facilitating peristaltic transit [21,22]. Through these mechanisms, the gut microbiota influences the immunoinflammatory response, energy metabolism, and neuroendocrine communication, thereby playing a decisive role in overall human health [23,24]. An imbalance of the gut microbiota (dysbiosis) has been associated with increased susceptibility to gastrointestinal [25,26,27], metabolic [28,29], cardiovascular [30,31], neurodegenerative [32] and autoimmune diseases, in addition to cancer [33,34]. Exposure to pesticides induces significant changes in the abundance of various microbial taxa in relation to the alteration of the gastrointestinal microbiota. This phenomenon is characterised primarily by the proliferation of opportunistic pathogens at the expense of beneficial and resident bacterial genera such as *Lactobacillus* and *Bifidobacterium*, resulting in intestinal dysbiosis [35,36]. This alteration has been extensively documented in animal models exposed to organophosphates. Similarly, research on humans exposed to organophosphates indicates that exposure to environmental pesticides modifies not only the taxonomic composition, but also the metabolic pathways of bacteria. These modifications affect basic cellular processes and the biosynthesis of cellular compounds [37,38]. In terms of its impact on intestinal permeability and inflammation, pesticide-induced dysbiosis can compromise the integrity of the intestinal barrier. This decreases the expression of tight junction proteins and facilitates the translocation of microbial components, such as lipopolysaccharides (LPSs), into the bloodstream [39]. This increased intestinal permeability (dysbiosis) promotes the activation of systemic inflammatory pathways and low-grade chronic inflammation, which is central to the pathophysiology of metabolic disorders such as metabolic syndrome and type 2 diabetes, as well as cardiovascular disease [40]. Regarding the disruption of microbial metabolites and signalling, pesticide-induced alterations to the microbiota affect the profile of metabolites generated by it, such as short-chain fatty acids and other bioactive compounds. These metabolites regulate immune, metabolic and neuroendocrine functions [35]. Altered modulation of these metabolites may contribute to pro-inflammatory processes, insulin resistance, and systemic metabolic dysfunction (all of which are key factors in the development of chronic diseases) [35,41]. Microbial imbalance can contribute to both systemic inflammation and immune dysfunction [28,32] as well as toxicant-related pathologies [25]. In this context, the gut microbiota not only modulates digestive health, but mediates toxic responses to environmental xenobiotics by participating in their biotransformation and partial detoxification, an emerging area of significant interest in contemporary toxicology [29]. Numerous studies have documented the disruptive effects of organophosphate, carbamate and neonicotinoid pesticides on the intestinal epithelium and gut microbiota [9,42,43,44]. These agents can alter the structure of bacterial communities, change intestinal permeability and create a pro-inflammatory state linked to oxidative stress and mitochondrial dysfunction [45,46]. Such alterations are linked not only to an increased risk of chronic diseases in adults, but also to transgenerational effects, given that prenatal exposure can adversely affect foetal development through epigenetic and microbiological mechanisms [47,48,49].

Although regulatory measures have been implemented to reduce exposure levels, the persistence of certain organochlorine compounds and the continued use of pesticides in poorly controlled settings, remain significant public health concerns [50]. In this context, this article provides an overview of scientific evidence derived from systematic reviews of experimental and observational studies in humans and animal models, paying special attention to chronic diseases resulting from alterations in the gut microbiota and intestinal membrane permeability. The objective is to evaluate and synthesise available scientific evidence from systematic reviews of experimental and observational studies in humans and animal models, with a particular focus on chronic diseases associated with alterations in gut microbiota and intestinal membrane permeability. In addition, this review aims to provide an up-to-date and robust overview to guide future research and public health policies. The PICO framework was used to define the research question:

P (Population/Participants): Humans exposed to pesticides used in agricultural production, as well as animal models with genetic, biological or behavioural similarities to humans.

I (Intervention/Exposure): Acute or chronic exposure to pesticides through occupational, environmental or dietary contact.

C (Comparison): Human populations or animal models with no exposure or minimal exposure to pesticides.

O (Outcomes): Alterations associated with modifications in the gut microbiota, including changes in intestinal barrier permeability, and their relationship to the development of chronic diseases.

Thus, the research question was formulated as follows: What are the effects of exposure to agricultural pesticides on human health, and how are these effects mediated by alterations in the gut microbiome in comparable animal models and humans, according to available scientific evidence? To systematically delimit and organise the available scientific evidence on this topic, an overview of published systematic reviews covering the period 2018–2025 was undertaken. Systematic reviews provide a rigorous and objective summary of the existing scientific literature on a given topic, by integrating multiple individual studies into a single document, critically appraised by experts. The quality of the evidence was assessed based on the principles of evidence-based medicine and the guidelines of Reyna et al. [51], Galarza and Cruz [52], and Davidoff et al. [53]. Systematic reviews were prioritised for inclusion as they constitute one of the highest levels of scientific evidence. We verified that the reviews had clearly defined objectives, explicit search strategies, transparent inclusion criteria, and a structured synthesis of results. Although no formal risk-of-bias assessment tools were used, restricting inclusion to systematic reviews published in peer-reviewed journals served as an indirect approach to reducing potential methodological bias.

## 2. Methods

### 2.1. Research Design

A systematic review of the available scientific literature was conducted, in accordance with the PRISMA (Preferred Reporting Items for Systematic Reviews and Meta-Analyses) Guidelines, to ensure transparency, reproducibility, and methodological rigour in the identification, selection, and analysis of studies.

The process was carried out in several clearly defined phases. First, an exhaustive search of relevant scientific databases was carried out using keywords and MeSH terms related to pesticides, human and animal exposure, and their associated effects on health. Systematic reviews were then selected based on predefined inclusion and exclusion criteria, focusing on studies that provided direct evidence of the relationship between pesticide exposure and gut microbiota alterations, including changes in intestinal barrier permeability and their association with disease development.

Once the systematic reviews had been selected, relevant data were extracted, including information about the pesticide, study design, population, health alterations, outcome variables, main findings, and conclusions.

Finally, the results were synthesised systematically to provide a comprehensive and objective assessment of the impact of pesticides on human and animal health, and to inform recommendations for public health measures aimed at preventing and mitigating diseases associated with pesticide exposure and consumption.

### 2.2. Search Strategy

A comprehensive search of the scientific literature published between 2018 and the present day was conducted using the Medline/PubMed, Embase and Web of Science databases. The aim was to compile the most relevant information available on the relationship between exposure to pesticides and health. To ensure consistency in the evaluation and analysis of the evidence, only full-text systematic reviews published in English were included.

Initially, the search was conducted by combining key concepts with the Boolean operators AND and OR. The initial search algorithm was established with the terms (“pesticides”) AND (“disease” OR “illness”) AND (“gut microbiota”). This strategy generated many results (10.850), although they were very heterogeneous in relation to the topic of interest. Finally, the search was refined to focus specifically on the relationship between pesticides and gut microbiota, using the final syntax: (“pesticides”) AND (“gut microbiota”). Table 1.

To ensure the highest quality of scientific evidence, only systematic reviews corresponding to Level I evidence were included. These reviews integrate the findings of all relevant randomised clinical trials and have been critically reviewed by experts in the field.

The review was conducted in two phases; from June to late September 2024, and from January to late May 2025. A systematic and structured procedure was followed to ensure the results obtained were comprehensive and valid. Previous searches were conducted at different times and served as the basis for the searches performed during the periods indicated above, following the final decision to undertake the study presented in this manuscript. This systematic review was registered in the Open Science Framework (OSF) on 20 December 2025 (registration number: osf.io/jruh5).

### 2.3. Eligibility Criteria

The inclusion criteria considered only systematic reviews comprising observational studies in humans and experimental studies in animal models relevant to humans (mainly rats and mice). Only articles written in English with full text availability were included. Systematic reviews specifically addressing pesticide-induced alterations in gut microbiota and intestinal barrier permeability associated with chronic diseases were considered eligible.

Systematic reviews addressing persistent organic pollutants, metals, endocrine disruptors, and plastic substances were excluded as were reviews of other substances not used in traditional agriculture. Reviews including experimental studies on birds or animals with characteristics that differ substantially from humans, were also excluded.

### 2.4. Article Selection

First, an initial search was conducted using the algorithms described in the ‘Search Strategy’ section above. The aim was to estimate the volume of existing publications and compile the most relevant information to answer the research question.

Next, the articles relevant to this study were identified using the established inclusion and exclusion criteria. This phase involved reviewing titles and abstracts to determine the relevance of each publication.

Following this, a more detailed screening was carried out by reviewing the full text of the preselected articles.

Each paper was assessed for suitability, and those that did not align with the central theme of the review or lacked relevant conclusion, were excluded.

MJDD and IOS performed the initial screening. Both reviewers independently assessed the titles and abstracts of all articles retrieved from the electronic search. To minimise selection bias, they worked independently, without knowledge of each other’s assessments.

MJDD and TRR performed the full-text screening. The full texts of the preselected articles were reviewed independently by both evaluators, each working without access to the other’s assessment. Discrepancies were resolved through consensus discussion.

Finally, the studies included in the review were selected following the methodological recommendations of Galarza and Cruz (2024) [52] to ensure rigour and consistency in the selection process. The three researchers discussed and reached consensus on the final studies to be included, based on the predefined inclusion and exclusion criteria. At this stage, independent review was unnecessary, since the goal was to achieve unanimous agreement on study inclusion.

### 2.5. Data Extraction

The following variables were considered relevant and extracted from the selected articles: authors, year of publication, pesticide, sample and/or type of study, results and conclusions. Only information pertinent to the review objectives was considered, and duplicated findings were avoided.

## 3. Results

### 3.1. Results of the Study Selection

The initial search using the algorithm (“pesticides”) AND (“disease” OR “illness” OR “gut microbiota”) produced 10.850 results. This was then refined using the syntax “pesticides” AND “gut microbiota”, reducing the number of studies identified to 350 articles. After applying the previously described eligibility criteria, 22 studies were selected for review.

**Figure 1:**
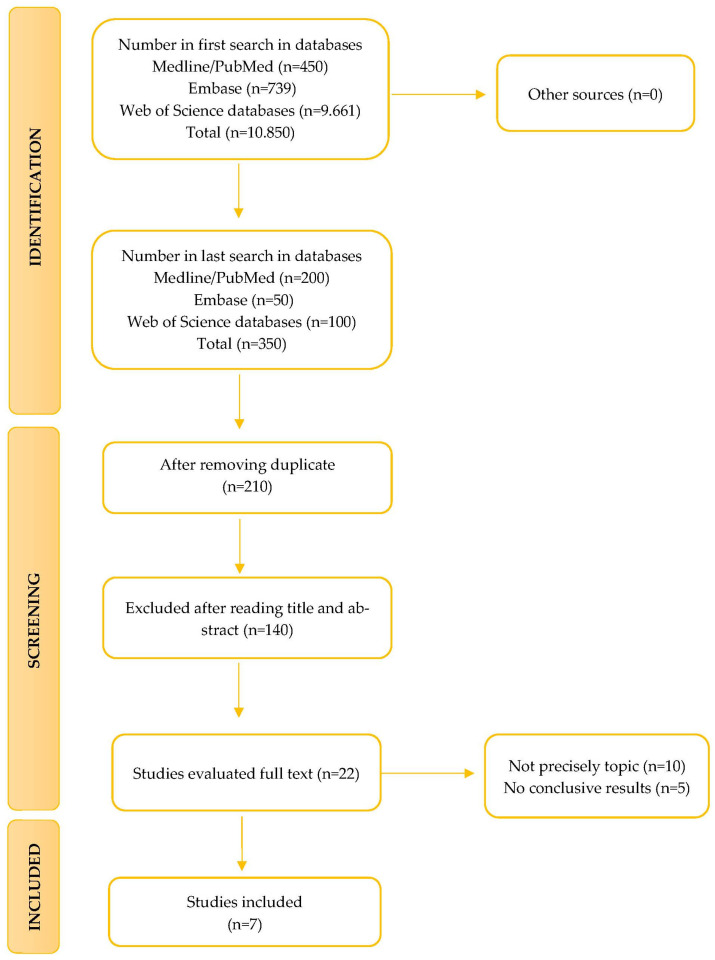
Flowchart for study selection.

The selected articles were then screened in detail, resulting in the exclusion of 10 that did not precisely match the theme of this review, and 5 studies that lacked conclusive results. Consequently, seven articles were included in the final systematic review. The entire selection process is illustrated in the corresponding flow chart (Figure 1).

Of the seven studies selected, three were conducted in the United States and four in Europe. The main characteristics and most relevant data from these studies are presented in Table 2.

### 3.2. Results of the Effects of Pesticide Exposure in Humans

Research conducted on human populations has shown that exposure to pesticides during pregnancy can have adverse effects on foetal development, including, among other things, a decrease in birth weight. A significant association has also been identified between pregnant women living less than 2.000 m from areas where these compounds are used, and an increased risk of their offspring developing autism spectrum disorders [55]. This risk intensifies depending on the degree of exposure, being particularly notable during the second and third trimesters of pregnancy, with an estimated increase of 30% [55]. Similarly, an inverse relationship has been described between prenatal exposure to pesticides and child neuropsychological development, as seen by the appearance of autistic traits in 11-year-old children who were exposed in utero [55].

Furthermore, scientific evidence indicates that exposure to these chemicals is associated with various metabolic disorders, such as insulin resistance, type 2 diabetes mellitus, dyslipidaemia, dysfunction in the biotransformation of chemical compounds, atherosclerosis, obesity and hepatic metabolism disorders [40,57].

Likewise, the existence of intestinal dysbiosis linked to the accumulation of adipose tissue has been documented. Added to this is the activation of the inflammatory response, mediated by the release of pro-inflammatory cytokines, together with alterations in the functionality of the immune system.

Regarding the intestinal microbiome, pesticide exposure has been seen to induce microbial imbalances and dysbiosis, increasing intestinal barrier permeability and generating multiple adverse effects on the health of individuals [40,54,55,56,57,58,59].

### 3.3. Results of the Effects of Pesticide Exposure in Animal Models

#### 3.3.1. Effects of Exposure in Rats

Experimental studies in rats have revealed a wide range of physiological alterations resulting from exposure to these substances. Nervous system alterations included an increase in the activity of excitatory pathways, a decrease in inhibitory activity, and a loss of dopaminergic neurons in the substantia nigra [55,56]. At the endocrine and metabolic level, elevated basal insulin levels have been documented, accompanied by liver dysfunction affecting key processes such as lipogenesis, gluconeogenesis and glycogenolysis, resulting in hepatotoxicity [40]. Likewise, increases in body weight, obesity, a higher prevalence of phenotypes compatible with type 2 diabetes mellitus, and alterations in the pancreatic islets, characterized by lipid accumulation, have been observed [55,58].

From an immunological perspective, exposure to these compounds is associated with a decrease in immune response and alterations in the mechanisms regulating inflammation. At the intestinal level, both structural and functional changes have been described, including micro- and macro-anatomical alterations located in the right intestine, a reduction in beneficial bacterial genera, the proliferation of potentially pathogenic species, and the establishment of a state of dysbiosis in the intestinal microbiota. Furthermore, the results indicate sex-specific differences in response to exposure, observed across all doses evaluated [54,58].

Collectively, these findings suggest that exposure to pesticides could compromise the homeostasis of the nervous, endocrine, immune, and gastrointestinal systems, underlining the importance of further investigation into their effects on human health and the potential extrapolation of these findings to humans.

#### 3.3.2. Effects of Gestational Exposure to Pesticides on Rat Offspring

Research conducted on rat pups whose mothers were exposed to pesticides during pregnancy has revealed multiple metabolic, neurological and developmental abnormalities [54,58]. Metabolically, hyperlipidaemia and hyperglycaemia were observed in female offspring, while persistent neurological dysfunction was observed throughout their lifespan [54]. In male foetuses, changes in gene transcription in the external genitalia have been reported, along with an increased susceptibility to prostate disease, obesity and kidney disease [40].

The development of the digestive system was also compromised, with delayed maturation of the gastrointestinal tract, dyskinesia and the onset of various intestinal diseases [59]. With respect to the reproductive system, ovarian alterations and abnormalities during the birthing process were documented [54]. At the neuroinflammatory and cellular level, prenatal exposure was also associated with changes in the inflammatory response and in the expression of genes involved in oxidative stress, particularly in brain regions such as the cortex and cerebellum [54,55,58].

Similarly, behavioural changes were identified in offspring, especially in their responses to novel situations, presenting exacerbated phenotypes like those seen in autism spectrum disorder [55]. Collectively, these findings strengthen the evidence that prenatal exposure to pesticides can have persistent adverse effects on offspring development and physiological function.

#### 3.3.3. Effects of Exposure on Mice

Research conducted in mice has shown that pesticide exposure induces significant alterations in the composition and function of the gut microbiome, characterized by dysbiosis and increased intestinal barrier permeability. In the metabolic sphere, impaired energy metabolism has been observed, including reduced weight gain in both sexes, lower body weight in females, and abnormal bacterial translocation [40]. Additionally, increases in blood glucose, glucose intolerance, hepatic lipid accumulation and alterations in the lipid profile have been reported, with raised triglyceride, total cholesterol, HDL and LDL levels [40].

From a genetic and microbiological perspective, exposure to these compounds affects quorum sensing regulation, promoting an increase in bacterial motility and pathogenicity, as well as modifications in the expression of genes linked to cell wall structure and pesticide biotransformation [59]. Similarly, alterations in enterohepatic metabolism, variations in bile acids, insulin resistance and heightened systemic inflammatory processes have been documented, all of which are associated with an increased risk of developing cardiovascular disease and colitis [55,59].

With regard to the central nervous system, dysregulation in the synthesis and release of neurotransmitters has been observed, along with neuronal inflammation, impaired locomotor activity, alterations in short-term memory, and behaviours similar to those seen in autism spectrum disorder [55,56].

In terms of body composition, males show greater susceptibility to abnormal translocation, as well as a significant increase in the accumulation of body, hepatic and epididymal fat [54,55,57,58]. Increases in serum triglyceride and glucose levels have also been reported, along with exacerbated glucose intolerance [40]. At the same time, alterations in gene expression related to metabolic pathways and glucose regulation, have been identified [40].

As a whole, all these findings indicate that pesticide exposure has a multi-systemic impact, compromising energy metabolism, immune homeostasis and neurological function, with effects that differ according to sex and duration of exposure.

## 4. Discussion

This systematic review, developed in accordance with the PRISMA principles, summarises and contrasts the available evidence on the effects of exposure to agricultural pesticides on gut microbiota and intestinal barrier permeability, as well as their relationship with chronic disease development. The review also considers the implications of prenatal exposure in the short and long-term. Analysing the reviews by Yue et al. [54], Yang et al. [55], Utembe and Kamng’ona [56], Yuan et al. [57], Gambarte and Wolansky [58], Djekkoun et al. [40] and Meng et al. [59], together with the other included literature, allows us to identify consistent patterns, methodological limitations and gaps in knowledge that are relevant for guiding future research.

This study aimed to evaluate and synthesise existing scientific evidence from observational studies in humans and experimental research in animal models relevant to humans. It focused on chronic diseases linked to changes in the gut microbiota and intestinal permeability due to exposure to agricultural pesticides.

Analysis of the selected studies reveals a significant degree of alignment on four key findings. Firstly, alterations in the gut microbiota, intestinal dysbiosis and increased intestinal permeability appear to be central mediators linking environmental exposure to systemic disease. Secondly, pesticide exposure is associated with low-grade chronic inflammation and oxidative stress. Thirdly, a consistent relationship has been found between pesticides and metabolic dysfunction, including insulin resistance, dyslipidaemia, and non-alcoholic fatty liver disease. Lastly, prenatal exposure to pesticides has been linked to lower birth weight and an increased risk of neurobehavioural alterations, including autism spectrum disorder.

From a metabolic perspective, the synthesised evidence substantially agrees on the idea that intestinal dysbiosis and increased intestinal permeability are central mechanisms linking pesticide exposure to insulin resistance, obesity, type 2 diabetes, and dyslipidaemia, as identified by Yue et al. [54], Yang et al. [55], Yuan et al. [57], Meng et al. [59], Djekkoun et al. [40], and Gambarte and Wolansky [58]. These authors concur that pesticides reduce the number of bacteria that produce short-chain fatty acids, particularly butyrate. This compromises the integrity of the intestinal barrier and promotes the translocation of bacterial endotoxins, activating low-grade systemic inflammation, thereby leading to metabolic alterations. This aligns with classic studies by Cummings et al. [60] and Flint et al. [61] on short-chain fatty acids which emphasise the vital role of these microbial metabolites in maintaining intestinal and metabolic homeostasis. Similarly, Yuan et al. [57] and Meng et al. [59] suggest that pesticides alter bile acid metabolism and FXR and TGR5 receptor signalling, thereby integrating the microbiota–gut–liver axis into metabolic pathogenesis.

Regarding hepatic and cardiovascular alterations, the reviews by Yuan et al. [57], Meng et al. [59] and Djekkoun et al. [40], notably align, agreeing that exposure to pesticides contributes to structural and functional alterations in hepatic parenchyma and vascular endothelium. At the hepatic level, evidence suggests increased lipid accumulation in hepatocytes, oxidative stress, and activation of inflammatory pathways that facilitate the progression of steatosis to non-alcoholic fatty liver disease. At the same time, a systemic pro-inflammatory state is observed in the cardiovascular system, which can compromise endothelial integrity, encourage vascular dysfunction, and favour atherogenic processes. These alterations appear to be interconnected via the gut-liver-vascular axis, where dysbiosis and increased intestinal permeability trigger an inflammatory chain reaction that can affect multiple organs.

The association observed between gestational exposure and lower birth weight, aligns with findings reported by Yang et al. [55] and Meng et al. [59], and is further supported by Yuan et al. [57], who review the link between low birth weight and an increased risk of cardio-metabolic diseases in adulthood. This suggests that the effects of prenatal exposure could extend beyond the neonatal period, influencing how metabolic risks develop and change throughout the life cycle.

The findings of Yue et al. [54] and Yuan et al. [57], regarding metabolic alterations related to prenatal exposure, agree that pesticide exposure is associated with intestinal dysbiosis, increased intestinal permeability and low-grade systemic inflammation. This gives rise to a central axis of physiological mechanisms capable of explaining adverse metabolic effects.

In terms of neurobehavioural effects, studies by Yang et al. [55] and Yue et al. [54], suggest that exposure during pregnancy is linked to intestinal dysbiosis and changes to the gut–brain axis. This increases the likelihood of characteristics and diagnoses associated with autism spectrum disorder, particularly when exposure occurs during the second and third trimesters. These findings are supported by murine models, which show neuroinflammation, GABAergic alterations and changes in dopaminergic circuits, as well as modifications in fatty acid and retinol metabolism in offspring [62].

The main strength of this study is how it brings together and compares evidence from both human studies and animal models in a systematic way. The multidisciplinary approach covers metabolic, immunological, neurological and gastrointestinal aspects, and includes a comparative analysis of key authors and the complementary literature. It also evaluates the prenatal and long-term effects, considering plausible biological mechanisms, with a particular focus on chronic diseases associated with alterations in the gut microbiota and intestinal permeability induced by exposure to agricultural pesticides. Furthermore, by focusing on the interaction between dysbiosis, decreased short-chain fatty acids, systemic inflammation, and metabolic dysfunction, this study provides an up-dated conceptual framework that allows critical periods of vulnerability to be identified. This approach enhances the validity of the conclusions and offers a comprehensive view of the systemic effects of pesticide exposure, thereby reinforcing the review’s relevance for future research, the development of prevention strategies and the creation of environmental health policies.

However, even with a systematic method and an integrative approach, there are still some limitations that need to be discussed. Firstly, there is insufficient human evidence to establish definitive causal relationships, given that many studies are cross-sectional or lack longitudinal follow-up. Secondly, there is significant variation in how the studies were performed, especially regarding the different types of pesticides evaluated. Thirdly, most studies struggle to separate the effects of pesticide mixtures from those of individual compounds, which complicates the interpretation of specific results. These limitations highlight the need for prospective studies with repeated exposure measurements, a longitudinal design and an integrated, multi-omic approach, to more accurately characterise the chronic effects of pesticide exposure on health and the underlying biological mechanisms.

## 5. Conclusions

This systematic review demonstrates that exposure to pesticides primarily impacts human health by altering the gut microbiome and intestinal permeability, thereby triggering systemic inflammation, metabolic dysfunction, and neurobehavioural effects. These underlying changes are crucial for the development of metabolic, inflammatory and neurodevelopmental disorders, particularly when exposure occurs during critical periods of prenatal development. Even though the studies mainly rely on animal models and vary in their methods, the alignment between human and experimental studies supports the relevance of environmental pesticide exposure as a preventable risk factor. Our analysis highlights the need to implement comprehensive public health strategies aimed at reducing exposure to pesticides, particularly among vulnerable groups such as pregnant women and children. This calls for a move towards more sustainable agricultural practices, better regulation and control of these hazardous chemicals, and the promotion of educational programmes to ensure their safe use. At the same time, it is essential to improve environmental surveillance systems, population bio-monitoring, and closely monitor maternal and child development to aid the early detection and mitigation of potential adverse health effects.

These results emphasise the importance of adopting a multidisciplinary approach that combines prevention, surveillance, and translational research to safeguard human health and lessen the burden of disease linked to pesticide exposure.

## Figures and Tables

**Table 1:** Search algorithms and number of articles in each database.

Database	Search Terms	Results
Medline/PubMed	(“pesticides”) AND (“disease” OR “illness”) AND (“gut microbiota”) (“pesticides”) AND (“gut microbiota”)	450 200
Embase	(“pesticides”) AND (“disease” OR “illness”) AND (“gut microbiota”) (“pesticides”) AND (“gut microbiota”)	739 50
Web of Science	(“pesticides”) AND (“disease” OR “illness”) AND (“gut microbiota”) (“pesticides”) AND (“gut microbiota”).	8.880 100

**Table 2:** Extraction of Relevant Data from the Systematic Review Articles: Main Results and Conclusions.

Author Year	Pesticide	Sample and/or Type of Study	Results	Conclusions
FINDINGS IN HUMANS				
**Yue, Y. et al.,** **2024** [54]	Insecticide (β-HCH)	Human mothers	Weight loss in offspring.	This study concludes that prenatal exposure to pesticides is associated with alterations in gut microbiota, generating a state of dysbiosis that can negatively affect the development, metabolic health, and behaviour of human offspring. These microbial modifications appear to influence key metabolic pathways related to energy regulation and body growth. Low body weight and modifications in bacterial genes associated with carbohydrate and lipid metabolism have been identified, as well as behavioural abnormalities in offspring. These findings highlight the importance of investigating the effects of pesticides on the microbiome and their role in the development of metabolic and neurological diseases.
	Insecticide (Mecarbam)	Human mothers	Weight loss in offspring.	
**Yang, Y. et al.,** **2023** [55]	Glyphosate	Case–control study of pregnant women	Increased risk of autism spectrum disorder in offspring of pregnant women living less than 2000 m from places where herbicides are used.	This study establishes a link between alterations in the gut microbiome and autism spectrum disorder. Exposure to pesticides causes dysbiosis in gut microbiota and neurodevelopment, contributing to neurological defects and behavioural alterations. In summary, dysbiosis of the gut microbiome is a crucial factor in the symptoms of autism spectrum disorder related to pesticide exposure.
	Chlorpyrifos	Case–control study of pregnant women	Exposure in mothers positively correlates with autism spectrum disorder in offspring.	
		Cohort study pregnant women	Inverse association between prenatal exposure and domain-specific neuropsychological development in children at 12 months.	
		Cohort study pregnant women	Increased autistic traits in 11-year-old children with prenatal exposure.	
		Case–control study of pregnant women	Increased risk of autism spectrum disorder in offspring of mothers exposed during the second or third trimester of pregnancy.	
		Case–control study of pregnant women	Higher risk of autism spectrum disorder with greater exposure during pregnancy.	
	Pyrethroids	Case–control study of pregnant women	Exposure during the third trimester was associated with an increased likelihood of presenting symptoms of autism spectrum disorder.	
		Cross-sectional study of pregnant women	Higher levels of pyrethroids in urine were associated with an increased risk of autism spectrum disorder in offspring.	
		Case–control study pregnant women	Prenatal exposure was associated with an increased risk of autism spectrum disorder.	
	Imidacloprid	Case–control study pregnant women	Increased risk of autism spectrum disorder in offspring in 30% of cases.	
**Utembe, W.; Kamng’ona, A.W.** **2021** [56]	Glyphosate. Chlorpyrifos. Pyrethroids. Imidacloprid. Diazinon. Ammonium glufosinate.	Case–control studies in humans. Cohort studies in humans.		This study confirms the presence of cognitive deficits, stress-related hyper-mobility, social interaction dysfunction, reduced responsiveness to social novelty in adults and short-term memory impairment. Acetylcholinesterase activity was altered, implying enzyme inhibition and hypo- or hypersensitisation of cholinergic and GABAergic systems. There is also increased oxidative stress, which promotes ageing and chronic diseases.
**Yuan, X. et al.,** **2019** [57]	Glyphosate. Chlorpyrifos. Monochrotophos. Pyrethroids. Imidacloprid. Diazinon. Glufosinate ammonium. Propamocarb.	Human body fluids. Pregnant women. Double-blind, placebo-controlled, randomised study in patients with type 2 diabetes. Case–control study in humans.	A decrease in the number of beneficial bacteria (*Bifidobacterium* and *Lactobacillus*), accompanied by an increase in the number of *Enterococcus* and *Bacteroides*. This can change the pH and affect the production of short-chain fatty acids in the intestine, facilitating the colonisation of potentially pathogenic bacteria. In addition, the intestinal barrier is affected, leading to the release of the chemokine IL-8 and causing inflammation.	This article suggests that changes in the gut microbiome and metabolites can cause adverse effects in the host, in the transduction of extracellular signals from the plasma membrane to the cell and along the intracellular chain to stimulate the cellular response. Different bile acids can bind to different receptors, which can lead to atherosclerosis, hepatic lipid metabolism disorders, and dysbiosis due to fat accumulation. The alteration of the intestinal microbiome balance and increased intestinal permeability due to pesticide absorption are potential risk factors for increasing the entry of molecules with higher molecular weight, leading to an inflammatory response and, ultimately, low-grade inflammation. Innate immune cells activated by endotoxic bacteria can release various pro-inflammatory cytokines, inducing low-grade inflammation and even neuronal inflammation. In short, pesticides can act on intestinal microbes, affect their metabolites, and destroy the mucosa and intestinal cells. These changes cause pathological changes by acting on receptor sites in different tissues and organs.
**FINDINGS IN ANIMAL MODELS**				
**Yue, Y. et al.,** **2024** [54]	Herbicide (Ammonium glufosinate)	Father mice	Behavioural abnormalities.	This study concludes that, in animal models, exposure to environmental disruptors and/or adverse experimental conditions during the prenatal and early stages of development, induces lasting systemic effects that affect metabolism, the nervous system, the endocrine system and the gut microbiota in a coordinated manner. These effects vary according to sex and developmental stage. Metabolically, exposed offspring presented consistent alterations in energy regulation, including obesity, hyperlipidaemia, and glycaemic disorders (hyperglycaemia and hypoglycaemia in females), accompanied by persistent changes in body weight into adulthood. These effects were associated with disruptions in the gut microbiota, characterized by early dysbiosis and profound alterations to the cecal microbiome, and evidence of bacterial translocation to the liver and spleen. This suggests a loss of intestinal barrier integrity and a systemic pro-inflammatory state. Changes were also observed in genes related to inflammation and oxidative stress in the cortex and cerebellum, indicating the central nervous system’s vulnerability to the altered metabolic environment. In the behavioural sphere, exposed animals exhibited behavioural abnormalities, particularly in response to novel situations, along with alterations in glutamatergic function and GABAergic signalling in the amygdala. These findings suggest disruption of neuronal development, leading to neuro behavioural deficits and abnormal neurodevelopment throughout life, with effects that were often sex-specific or more pronounced in one sex. Furthermore, neurodevelopmental defects were documented in males, accompanied by progressive auditory alterations, reinforcing the concept of chronic neurosensory impairment. From an endocrine and reproductive perspective, exposure was found to alter multiple hormonal axes. This was evidenced by increased LH levels, morphological changes to the uterus (such as increased intrauterine weight and luminal epithelial height), modifications to gene transcription in the male external genitalia, and a reduced sperm count. These changes were associated with an increased risk of reproductive and systemic pathologies, including prostate, ovarian and kidney diseases, congenital anomalies, and reduced birth rates. These findings emphasise the importance of investigating the effects of pesticides on the microbiome and their role in the development of chronic diseases.
	Insecticide (Combination of Boscalid, Captan, Chlorpyrifos, Thiacloprid, Thiophanate, and Ziram)	Father mice	Obesity and metabolic disorders.	
	Insecticide (Chlorpyrifos)	Rat offspring	Hyperlipidaemia and hyperglycaemia in female offspring. Changes in rat behaviour when faced with new situations. Changes in glutamine function and GABA signaling in the amygdala.	
		Mouse offspring	Interference with developing neurons.	
	Insecticide (Nitenpyram)	Parent mice	Decrease in serum glucose in female offspring.	
	Fungicide (Procymidone)	Progenitor rats	Metabolic disorders. Neurological impairments throughout life.	
		Parent mice	Metabolic disorders. Neurodevelopmental disorders in sex-dependent offspring.	
	Insecticide (Fenvalerate)	Parent mice	Increased intrauterine wet weight. Increased height of luminal epithelial cells. Increased LH.	
	Insecticide (Dichlorodiphenyltrichloroethane)	Parent mice	Neurodevelopmental defects in male mice.	
	Insecticide (Cypermethrin)	Parent mice	Hearing impairments that develop slowly over time.	
	Fungicide (Triticonazole)	Progenitor rats	Alteration of endocrine effects. Changes in the genome transcription of the external genitalia of the male foetus.	
	Fungicide (Flusilazole)	Progenitor rats	Alteration of endocrine effects.	
	Insecticide (Chlordecone)	Parent mice	Defects and reduction in sperm count.	
	Herbicide (Glyphosate)	Progenitor rats	Prostate disease, obesity, kidney disease, ovarian disease, and birth abnormalities. Changes related to inflammation and oxidative stress genes in the cortex and cerebellum of offspring.	
	Herbicide (paraquat)	Baby mice	Increased weight in adults among descendants.	
	Herbicide (ammonium glufosinate)	Parent mice	Abnormal behaviour.	
	Insecticide (permethrin)	Baby mouse	Negative impact on gut microbiota	
	Insecticide (endosulfan)	Parent mice	Metabolic disorders. Obesity.	
	Insecticide (chlorpyrifos)	Parent rats	Bacterial translocation in the liver and spleen. Lower birth rate. Negative impact on gut microbiota. Profound changes in the microbiome of the caecum	
		Mouse offspring	Negative impact on gut microbiota. Dysbiosis at an early age in the intestinal membrane.	
		Rat offspring	Microbiome modifications. Hyperlipidaemia in female offspring. Hypoglycaemic alterations in female offspring. Alterations in rat behaviour in new situations. Reduced immune response in females. Asthma.	
	Insecticide (Nitenpyram)	Parent mice	Decrease in blood glucose in female offspring.	
**Yang, Y. et al.,** **2023** [55]	Glyphosate	Sprague-Dawley rats	Changes in maternal behaviour. Changes in neural plasticity. Changes in neural plasticity. Perinatal exposure leads to changes in the behaviour of rat offspring.	This study shows that pesticide exposure, especially during the prenatal and perinatal periods, alters the gut microbiota (dysbiosis) and neurobiological development, contributing to neurological and behavioural deficits similar to those observed in autism spectrum disorders. In behavioural terms, early exposure was associated with persistent deficits in social interaction, communication and exploration of novelty, alongside repetitive behaviours, hyper-mobility, increased stress reactivity, as well as cognitive and motor impairment. At the neurobiological level, changes in neuronal plasticity, morphological alterations in glial cells, neural and systemic inflammation, loss of dopaminergic neurons, and dysregulation of the GABAergic and cholinergic systems were observed, suggesting an excitation-inhibition imbalance. Changes in blood–brain barrier permeability and pituitary hormone release, were also detected, indicating disruption of the neuroendocrine axis. In addition, exposure affected maternal behaviour, which may have enhanced the negative effects on the socio-emotional development of the offspring.
		ddY mice	Cognitive impairment. Social interaction impairment. Behavioural abnormalities like autism spectrum disorder in the offspring of male mice.	
		Swiss mice	Deficit in social interaction. Repetitive stereotypical behaviour. Morphological changes in glial cells residing in the brain. Reduction in blood–brain barrier permeability. Alteration in acetylcholinesterase activity.	
	Chlorpyrifos	Sprague-Dawley rats	Existence of behaviours typical of autism spectrum disorder phenotypes (impaired social communication and confined and repetitive behaviour).	
		BTBR mice	Exposure during prenatal development promotes the existence of behavioural traits typical of autism spectrum disorder, including impairments in social and communication domains (alterations in ultrasonic vocalization and high levels of repetitive behaviours).	
		Wistar rats	Hypermobility and stress-related hypermobility. Hypo- or hypersensitisation of the cholinergic and GABAergic systems. Increased transcription of the GABA-A-A2 subunit and M2 receptor genes. Inhibition of acetylcholinesterase activity. Stimulation of pituitary hormone release. Systemic inflammation (TNFR). Decreased responsiveness to social novelty in adulthood. Communication deficits similar to those seen in autism spectrum disorder.	
		C57BL/6 mice	Exposure during prenatal development is associated with long-term negative effects on social behaviour and decreased exploration of unfamiliar items.	
		C57BL/6 mice	Exposure during prenatal development caused impairments in social behaviour and excitatory-inhibitory balance.	
		Fmr1-KO rats	Exposure during development led to exacerbation of a phenotype like autism spectrum disorder.	
	Pyrethroids	Wistar rats	Loss of dopaminergic neurons in the substantia nigra.	
		C57BL/6 mice	Neuronal inflammation.	
	Diazinon	C57BL/6 mice	Decreased regulation of neurotransmitters.	
	Glufosinate ammonium	ICR mice	Impaired motor activity. Behaviour similar to autism spectrum disorder. Impaired short-term memory formation.	
**Gambarte, P. C.K.; Wolansky, M. J.** **2022** [58]	Organophosphate insecticides (Chlorpyrifos)	Kunming male mice	Alterations to microbiota composition and metabolic pathways.	This study confirmed the existence of alterations in the gut microbiome that led to a reduction in beneficial bacteria and an increase in harmful bacteria, causing intestinal dysbiosis. Obesity, diabetes, alterations in the inflammatory response, genomic changes and morphological and functional changes in the intestine, have been identified as a consequence of pesticide use. Alterations in gene expression related to metabolic pathways, glucose intolerance, and pesticide biotransformation have also been demonstrated.
		Male Wistar rats	Alterations to microbiota composition. Increase in the abundance of opportunistic pathogens. Microbiota alterations are associated with obesity, diabetes phenotypes, and alterations in pancreatic islet cells. Alteration of the mechanism responsible for controlling the inflammatory response. Micro and macro structural alterations in the right intestine.	
	Organophosphate insecticides (Diazinon)	C57BL/6 mice	Changes in the composition of the gut microbiome. Changes in the functional metagenome and metabolic pathways. Differences are observed depending on the sex of the mouse.	
	Organophosphate insecticides (Monochrotophos)	Rats, CFT-Wistar	Changes in the composition of the gut microbiome. Functional and morphological changes in the intestine.	
		Mice, BALB/c	Changes in the composition of the gut microbiome. Changes in the expression of genes related to metabolic pathways, glucose intolerance and pesticide biotransformation.	
	Carbamate insecticide (Aldicarb)	C57BL/6 mice	Alterations to microbiota composition. Specific changes in the microbiome for each genus of bacteria during exposure.	
	Pyrethroid insecticide (Permethrin)	Male Wistar rats and lactating offspring	Alterations to microbiota composition. Reduction in beneficial bacterial genera and increase in non-beneficial bacterial genera compared to the control.	
	Systemic fungicide (Propamocarb)	ICR male mice	Changes in the composition of the gut microbiome 7 days after the start of oral exposure.	
	Fungicide Carbamate benzimidazole (Carbendazim)	ICR male mice	Alteration of the microbiome composition after 7 days. Increase in harmful bacteria and decrease in beneficial bacteria.	
		Male C57BL/6 mice	Alteration of the microbiome composition after 7 days. Increases and decreases in relative abundance depending on bacterial genus (beneficial or harmful).	
	Fungicide Triazole compounds (Epoxiconazole)	Female Sprague- Dawley rats	Alteration of the microbiome composition. Increases and decreases in relative abundance, depending on bacterial genus.	
	Herbicide Organophosphate (Glyphosate)	Sprague-Dawley rats	Gut microbiota dysbiosis and sex-dependent effects at all doses studied.	
	Herbicide Phenoxyacetic acid (2,4 D)	Male C57BL/6 mice	Increase and decrease in relative microbial abundance based on bacterial genus.	
**Djekkoun, N. et al.,** **2021** [40]	Organophosphates (Chlorpyrifos)	Rats	Higher number of harmful bacteria. Lower number of beneficial bacteria. Increase in potentially pathogenic flora. Decrease in beneficial flora. No impact or increase in body weight in adults. Low body mass and short body length at birth. Changes in plasma glucose levels and lipid profile. Significant difference in body weight.	This study has shown that exposure to pesticides modulates bacterial populations, impacting the health of the host. Intestinal dysbiosis induced by these compounds is associated with alterations similar to those observed in metabolic syndrome, where bacterial translocation, increased intestinal permeability and microbial dysmetabolism generate low-grade inflammation. These mechanisms contribute to imbalances in energy homeostasis and increase the risk of developing chronic inflammatory diseases. It has been demonstrated that various pesticides can induce endotoxaemia, mucosal permeability, and pro-inflammatory activation, leading to systemic inflammatory responses. Alterations in microbial substrates in the intestine have also been observed, leading to changes in short-chain fatty acid profiles and altering energy collection through targeted dysbiosis, reinforcing their role in metabolic disruption and the development of chronic inflammatory pathologies. Pesticides alter the gut microbiome, influence energy metabolism and promote low-grade inflammation. They also impact on metabolic health and the development of chronic diseases.
		Mice	Increase in potentially pathogenic flora. Decrease in beneficial flora. Abnormal permeability.	
	Organophosphates (Diazinon)	Mice	Altered microbiome composition. Impaired energy metabolism. Male animals are more susceptible to abnormal translocation. Reduced body weight gain.	
		C57BL/6 mice	Increase in potentially pathogenic flora. Decrease in beneficial flora. Significant increase in body, liver and epididymal fat. Increase in serum TG and glucose levels.	
	Organophosphates (MCP)	Mice	Increase in potentially pathogenic flora. Increased blood sugar levels. Glucose intolerance.	
	Organochlorines (TCDF)	Rats	Decrease in beneficial flora. Inflammation and hepatic lipogenesis, gluconeogenesis, and glycogenolysis alterations.	
	Organochlorines (DDT)	Rats	Increase in potentially pathogenic flora. Decrease in beneficial flora.	
	Organochlorines (PCP)	Female mice	Increase in potentially pathogenic flora. Decrease in beneficial flora. Reduction in body weight.	
	Benzimidazoles (CBZ)	Mice	Increase in potentially pathogenic flora. Decrease in beneficial flora. Accumulation of liver lipids. Increase in TG, cholesterol, HDL, and LDL.	
**Utembe, W.; Kamng’ona, A. W.** **2021** [56]	Glyphosate. Chlorpyrifos. Pyrethroids. Imidacloprid. Diazinon. Glufosinate ammonium	Rats. Mice.	Alterations in maternal behaviour and repetitive, stereotypical behaviours consistent with autism spectrum disorder. Alterations in neuronal plasticity; morphological changes in glial cells; loss of dopaminergic neurons in the substantia nigra; neuronal inflammation. Decreased permeability of the blood–brain barrier. Alterations in brain levels of short-chain fatty acids, negative regulation of neurotransmitters and inhibition of acetylcholinesterase. Hypo- or hypersensitisation of the cholinergic and GABAergic systems, and increased transcription of the GABA_A_A2 subunit and M2 receptor. Stimulation of the release of pituitary hormones and inflammatory mediators (TNF-α). Increased oxidative stress is associated with ageing and chronic diseases.	This study demonstrates that exposure to pesticides along with gut microbiota alterations and dysbiosis, lead to significant alterations at multiple biological and functional levels. The effects observed encompass behavioural, neuroplastic and neuroanatomical dimensions, as well as changes blood–brain barrier integrity, providing evidence of a widespread impact on the central nervous system. Alterations in neurochemical regulation and neurotransmission systems were also identified, indicating disrupted neuronal communication. Furthermore, the findings reveal activation of neuroendocrine and inflammatory pathways, together with increased oxidative stress, suggesting the involvement of pathophysiological processes linked to chronic inflammation and ageing. As a whole, these alterations reflect an integrated and systemic impact that could contribute to the development of neurological and neurobehavioural disorders, reinforcing the importance of addressing these effects from both mechanistic and multidisciplinary perspectives.
**Meng, Z. et al.,** **2020** [59]	Organophosphate insecticide (Malathion)	Mice	Gut microbiota composition disorders. Altered genes involved in quorum sensing, increased motility, pathogenicity, and genes related to cell wall components.	This study demonstrates that exposure to pesticides can alter the composition of the gut microbiome, which, in turn, affects the production and function of its key metabolites. The affected metabolites include acetate, propionate, and butyrate—the principal short-chain fatty acids produced by the gut microbiota—which regulate microbiota function and host–microbiota interactions through several key mechanisms. Firstly, these short-chain fatty acids are primarily produced in the caecum and proximal colon, where they reach their highest concentrations. From there, they act not only locally in the intestine but can also enter the peripheral circulation via the portal vein, allowing them to exert systemic effects on various host tissues. This demonstrates that the metabolic activity of the gut microbiota has a direct impact beyond the intestine. Metabolic disorders, including insulin resistance and obesity, have been identified. Exposure to these substances can adversely affect health, especially the digestive system and metabolism. Evidence indicates that early disruption of digestive tract maturation impairs nutrient absorption and promotes weight gain and lipid accumulation. This imbalance in lipid metabolism generates inflammation, which interferes with bile acid metabolism and can cause enterohepatic disorders, increasing the risk of cardiovascular disease. In addition, chronic colon inflammation and liver toxicity aggravate overall health, reflecting metabolic disorders and alterations in digestive function. These effects highlight the importance of understanding the interactions between the gut microbiome, metabolism and environmental factors in the development of chronic diseases.
	Organophosphate insecticide (Diazinon)	Mice	Alterations to the gut microbiota. Metabolic profile disorders.	
	Organophosphate insecticide (Chlorpyrifos) Organophosphate insecticide (Chlorpyrifos)	Mice	Intestinal inflammation and abnormal permeability. Insulin resistance and obesity.	
		Rats	Delayed maturation of the digestive tract.	
	Organophosphate insecticide (Dichloro- diphenyltrichloroethane)	Rats	Weight gain and lipid accumulation.	
	Pyrethroid insecticide (Permethrin)	Baby rats	Dyskinesia and intestinal disease.	
	Benzimidazole fungicide (Carbendazim)	Mice	Intestinal microbiota disorders. Lipid metabolism disorder and inflammation.	
	Systemic fungicide (Propamocarb)	Mice	Lipid and bile acid metabolism disorders. Enterohepatic metabolism disorders and possible cardiovascular disease.	
	Systemic fungicide that inhibits Ergosterol (Imazalil)	Mice	Colon inflammation.	
	Systemic fungicide containing Triazole compounds (Epoxiconazole)	Rats	Hepatic toxicity.	
	Systemic fungicide composed of Triazole (Penconazole and its enantiomers)	Mice	Intestinal microbiota disorders. Metabolic profile disorders.	
**Yuan, X. et al.,** **2019** [57]	Glyphosate. Chlorpyrifos. Monochrotophos. Pyrethroids. Imidacloprid. Diazinon. Glufosinate ammonium. Propamocarb.	Mice. NOD mice. Adult male C57BL/6 mice. Sprague-Dawley rats. Randomized study in rats. Prospective randomized study, mother-baby pairs.	A decrease in the number of beneficial bacteria (*Bifidobacterium* and *Lactobacillus*), accompanied by an increase in the number of *Enterococcus* and *Bacteroides*. This can change the pH and affect the production of short-chain fatty acids in the intestine, facilitating the colonisation of potentially pathogenic bacteria. In addition, the intestinal barrier is affected, leading to the release of the chemokine IL-8 and causing inflammation.	This paper leads us to conclude that pesticides can alter the composition of the gut microbiome and its metabolites. Different bile acids can bind to different receptors, which can lead to atherosclerosis, hepatic lipid metabolism disorders, and dysbiosis due to fat accumulation. Short-chain fatty acids derived from microbial fermentation of fibre can inhibit histone deacetylases and serve as energy substrates by directly activating G protein-coupled receptors. The action of short-chain fatty acids on the GPR receptor in fat cells can cause dysbiosis due to fat accumulation. In addition, innate immune cells activated by endotoxic bacteria can release various pro-inflammatory cytokines, inducing low-grade inflammation and even neuronal inflammation. The alteration of the intestinal microbiome balance and increased intestinal permeability due to pesticide absorption are potential risk factors for increasing the entry of molecules with higher molecular weight, leading to an inflammatory response and, ultimately, low-grade inflammation. Furthermore, the action of short-chain fatty acids in the brain can affect appetite and is therefore associated with obesity and diabetes. In short, pesticides can act on intestinal microbes, affect their metabolites, and destroy the mucosa and intestinal cells. These changes cause pathological changes by acting on receptor sites in different tissues and organs.

## Data Availability

No new data were created or analyzed in this study. Data sharing is not applicable to this article.
